# SARS-CoV-2 testing and infection control strategies in European paediatric emergency departments during the first wave of the pandemic

**DOI:** 10.1007/s00431-020-03843-w

**Published:** 2020-10-13

**Authors:** Malte Kohns Vasconcelos, Hanna Renk, Jolanta Popielska, Maggie Nyirenda Nyang’wa, Sigita Burokiene, Despoina Gkentzi, Ewelina Gowin, Daniele Donà, Sara Villanueva-Medina, Andrew Riordan, Markus Hufnagel, Sarah Eisen, Liviana Da Dalt, Carlo Giaquinto, Julia A. Bielicki

**Affiliations:** 1 Paediatric Infectious Diseases Research Group, Institute for Infection and Immunity, St. George’s, University of London, London, UK; 2 Institute for Medical Microbiology and Hospital Hygiene, Heinrich Heine University Düsseldorf, Universitätsstr. 1, 40225 Düsseldorf, Germany; 3 Department of Paediatric Cardiology, Pulmonology and Intensive Care Medicine, University Children’s Hospital Tübingen, Tübingen, Germany; 4 Department of Children’s Infectious Diseases Medical University of Warsaw, Infectious Diseases Hospital in Warsaw, Warsaw, Poland; 5 Paediatric Department, University Hospital Lewisham, London, UK; 6 Clinic of Children’s Diseases, Institute of Clinical Medicine, Vilnius University, Vilnius, Lithuania; 7 Department of Paediatrics, Patras Medical School, University General Hospital of Patras, Patras, Greece; 8 Department of Health Promotion, Poznan University of Medical Sciences, Poznan, Poland; 9 Division of Paediatric Infectious Diseases, Department of Women’s and Children’s Health, University Hospital of Padua, Padua, Italy; 10 Pediatric Infectious Diseases Unit, Department of Pediatrics, Hospital Universitario 12 de Octubre, Madrid, Spain; 11 Department of Paediatric Infectious Diseases, Alder Hey Children’s Hospital, Liverpool, UK; 12 Division of Pediatric Infectious Diseases and Rheumatology, Department of Pediatrics and Adolescent Medicine, University Medical Center, Medical Faculty University of Freiburg, Freiburg, Germany; 13 Department of Paediatrics, University College London Hospital Foundation Trust, London, UK; 14 Pediatric Emergency Unit, Department of Women’s and Children’s Health, University Hospital of Padua, Padua, Italy; 15 Department of Infectious Diseases and Vaccinology, University of Basel Children’s Hospital (UKBB), Basel, Switzerland

**Keywords:** COVID-19, Preparedness, Survey, Triage

## Abstract

Between February and May 2020, during the first wave of the COVID-19 pandemic, paediatric emergency departments in 12 European countries were prospectively surveyed on their implementation of SARS-CoV-2 disease (COVID-19) testing and infection control strategies. All participating departments (23) implemented standardised case definitions, testing guidelines, early triage and infection control strategies early in the outbreak. Patient testing criteria initially focused on suspect cases and later began to include screening, mainly for hospital admissions. Long turnaround times for test results likely put additional strain on healthcare resources.

*Conclusion*: Shortening turnaround times for SARS-CoV-2 tests should be a priority. Specific paediatric testing criteria are needed.

**Table:** 

**What is Known:** • *WHO and public health authorities issued case definitions, testing and infection control recommendations for COVID-19 in January.* • *SARS-CoV-2 testing was made available across Europe in February.* **What is New:** • *Paediatric emergency departments implemented COVID-19-specific procedures rapidly, including case definitions, testing guidelines and early triage.* • *A third of surveyed departments waited more than 24 h for SARS-CoV-2 test to be reported, resulting in additional strain on resources.*

**What is Known:**

• *WHO and public health authorities issued case definitions, testing and infection control recommendations for COVID-19 in January.*

• *SARS-CoV-2 testing was made available across Europe in February.*

**What is New:**

• *Paediatric emergency departments implemented COVID-19-specific procedures rapidly, including case definitions, testing guidelines and early triage.*

• *A third of surveyed departments waited more than 24 h for SARS-CoV-2 test to be reported, resulting in additional strain on resources.*

## Background

European reference laboratories established widespread capacities for testing for severe acute respiratory syndrome coronavirus 2 (SARS-CoV-2) within a matter of days after a diagnostic test was made publicly available [1, 2]. World Health Organization (WHO) and public health authorities in Europe issued case definitions, testing and infection control recommendations for COVID-19 in January 2020.

The current understanding of COVID-19 in paediatric patients is that children more often have mild disease compared to adults [3, 4]. Current knowledge suggests that the peak of infectiousness of SARS-CoV-2 infection occurs a few days before and after the onset of symptoms, meaning that presymptomatic people are able to transmit the infection [5]. Transmission unobserved by public health authorities occurs frequently, with at least one-third of cases having been undetected during the early epidemic [6, 7].

The aim of this study was to describe the implementation of testing and infection control strategies and their evolution in paediatric emergency departments in Europe.

## Methods

From mid-February to the first week of May 2020, we surveyed all European paediatric collaboration sites within the PENTA ID paediatric research network weekly for developments in their testing and infection control strategies. Portable document format (pdf) survey forms were sent out by email to contact officers at 78 paediatric departments across Europe. The survey form is available as an online supplement to this article. Completed and signed survey forms were handed in by return email. Missing weekly replies were imputed as last observation carried forward.

## Results

Paediatric departments of 23 mostly tertiary care hospitals in 12 European countries (Belgium, Germany, France, Italy, Poland, Portugal, the UK, the Netherlands, Greece, Spain, Lithuania and Switzerland) participated in the surveys (response rate 29%). Multiple sites participated in the UK (5, 3 tertiary and 2 secondary level), Germany (5, 4 tertiary and 1 secondary level), Spain (3, 1 network of sites representing the Madrid region, 1 tertiary and 1 secondary level) and Poland (2, both tertiary level). In each of the remaining countries (Belgium, France, Italy, Portugal, the Netherlands, Greece, Lithuania and Switzerland), one site participated.

### Rapid implementation and evolution of standardised case definitions

By the end of February 2020, all hospitals had implemented standardised case definitions for suspected COVID-19 cases, with the majority (16 out of 21 participating at that point in time, 76%) following national government or public health authority guidelines and three directly following WHO guidelines. Standardised definitions of suspected cases showed high similarity between sites. All definitions consisted of a clinical component of acute respiratory infection and an epidemiological component of possible exposure to the virus. The latter changed between February and April: initially, definitions at all sites required contact within 14 days with a confirmed case or travel to specified geographic areas; in time, this changed to staying in any area with ongoing community transmission. Twenty participating sites used suspected case definitions from the beginning that did not exclude patients on detection of an alternative pathogen. Two of the Spanish sites and one site in Poland initially excluded patients with confirmed alternative diagnoses of respiratory infections from being suspect cases for COVID-19. This changed by April at all three sites, so that afterwards detection of another pathogen that could explain the respiratory symptoms no longer excluded a patient from being a suspect case and from undergoing SARS-CoV-2 testing.

### Strict testing guidelines in line with case definitions

Ten sites (43%) reported that they strictly only tested patients for SARS-CoV-2 if they matched the definition of a suspected case. Another 12 (52%) had a policy to only test patients matching the case definition but reported that exceptions occurred regularly. Until April, no site reported that their decision to test patients was based on separate local guidelines. In April, several German and UK sites started broader testing, first with testing of patients admitted to oncology or intensive care units and from the end of April with routine screening of all admitted patients. Table 1 shows example developments of testing guidelines at four participating children’s emergency departments.

**Table 1:** Examples of evolution of testing strategies in the context of national case and test numbers

		Tübingen	Warsaw	Lewisham	Padova
Date	Country, region	Germany, South	Poland, Central	UK, Greater London	Italy, North
Mid-February	Daily new cases/mil *Daily tests*	0.0 *No data available*	0.0 *No data available*	0.0 *459.3*	0.18 *No data available*
	Testing strategy	Patients with LRTI AND either returning from Wuhan (China) during the last 14 days OR contact to a SARS-CoV-2 positive person during the last 14 days Exception: in some highly suspicious cases, patients returning from other parts of China were tested as well or if the diagnosis of a contact person was highly suspected but not yet confirmed.	Patients with acute respiratory infection (sudden onset of at least one of the following: cough, sore throat, shortness of breath) AND, in the 14 days prior to onset of symptoms, met at least one of the following epidemiological criteria: Were in close contact with a confirmed or probable case of SARS-CoV-2 infection OR had a history of travel to areas with presumed ongoing community transmission of SARS-CoV-2 (China) OR attended a healthcare facility where patients with SARS-CoV-2 infections were being treated Close contact, probable/confirmed cases—according to ECDC definition	All children who were symptomatic with cough or fever returning from Northern Italy, Wuhan/China or Singapore or all countries initially affected by COVID-19	Fever (TC > 37.5 °C axillary) and/or respiratory symptoms (rhinitis, cough and dyspnoea) with close contact with a probable or confirmed case of COVID-19 within the previous 14 days and/or travelling in high-risk areas
Early March	Daily new cases/mil *Daily tests*	1.25 *No data available*	0.03 *No data available*	0.39 *1680.4*	11.23 *4115.7*
	Testing strategy	Acute respiratory symptoms with or without fever AND history of travel to areas with high risk of ongoing transmission or living in a high-prevalence area (different countries, adapted every few days by German Public Health Institute Robert-Koch-Institute) OR clinical or radiological signs of viral pneumonia without any alternative diagnosis	Epidemiological criteria changed: Had a history of travel to areas with presumed ongoing community transmission of SARS-CoV-2 (different countries)	Travel history to affected areas AND/OR Admitted with fever	Fever (TC > 37.5 °C axillary) and/or respiratory symptoms (rhinitis, cough and dyspnoea) and/or gastrointestinal symptoms (vomiting, diarrhoea) with or without close contact with a probable or confirmed case of COVID-19 within the previous 14 days
Early April	Daily new cases/mil *Daily tests*	66.78 *58,335.4*	7.51 *5424.9*	52.22 *9683.9*	75.99 *33,918.7*
	Testing strategy	Every symptomatic patient (fever OR respiratory symptoms OR diarrhoea OR loss of smell and taste) admitted to the children’s hospital, as well as immunosuppressed patients with fever and all patients undergoing HSCT	1. Every patient with fever, respiratory symptoms including sore throat, loss of smell and taste, malaise and rhinitis, diarrhoea, vomiting, abdominal pain, headache unless explained by another condition 2. Family members or close contacts of confirmed SARS-CoV-2 positive patients 3. Follow-up on confirmed SARS-CoV-2 positive patients (regular visits until two negative results of PCR from nasopharyngeal swab)	Any child who came in with fever and needed admission	As above
Early May	Daily new cases/mil *Daily tests*	11.46 *57,434.9*	8.59 *14,422.3*	69.44 *64,291.0*	21.12 *58,877.1*
	Testing strategy	All paediatric patients with one or more of the following symptoms (unless explained by another condition): Fever, respiratory symptoms including sore throat and rhinitis, loss of smell and taste, malaise, diarrhoea, vomiting, abdominal pain, headache A routine screening for in- and outpatients: - All admissions via paediatric A&E - All patients who need elective or non-elective surgery, intubation or sedation to perform different procedures - All patients admitted to PICU - All patients with cystic fibrosis - All patients on home-ventilation All high-risk patients (solid-organ-transplant, dialysis, immunosuppressed, haemato-oncology) who are admitted or seen regularly in the outpatient clinics are screened every 2 weeks	As above	Any child requiring hospital admission	As above

Daily new cases per million population; national numbers of daily confirmed new cases (all ages) and tests retrieved from ourworldindata.org/coronavirus, University of Oxford, based on ECDC reports [8]; number of tests for Germany retrieved from Robert-Koch-Institute situation report [9]; daily average of previous 7 days for: 23rd of February, 8th of March, 5th of April and 10th of May; *LRTI* lower respiratory tract infection, *HSCT* haematopoietic stem cell transplantation, *A&E* accident & emergency department, *PICU* paediatric intensive care unit

In terms of sampling site, 52% of the participating hospitals restricted testing to upper respiratory samples, while 48% obtained upper and lower respiratory samples if the latter could be obtained.

Although discharge and infection control strategies after admission relied heavily on test results, by the end of the survey period only 9 hospitals (39%) received test results multiple times daily, while another 7 (30%) had waiting times of more than 24 h before test results would be back. At all but two sites, where faster turnaround times could be achieved, expected time to test results did not change over the survey period. By the beginning of March, 9 (43%) were discharging patients with pending test results when they were clinically stable. Three of the seven hospitals where results took more than 24 h to come back would not discharge patients while test results were pending, regardless of whether the patients were clinically stable. By mid-March all hospitals were discharging patients with pending test results.

Until the beginning of March, sites saw up to 30 suspected cases per week. While only one site (in Germany) had a child positive for SARS-CoV-2, 67% of sites had already provided care to suspected cases of SARS-CoV-2. At the different sites, the highest number of patients tested per week differed widely between 7 per week for a secondary care hospital in Western Germany and 112 for a specialised tertiary care children’s hospital in North England. Community test centres for COVID-19 opened across Germany in early to mid-March and in the UK only in April. Opening of community test centres in proximity to the surveyed sites coincided with varying stages of development of overall case numbers in the areas. Therefore, our data allow no firm conclusion on whether opening of community test centres alleviated patient pressure on paediatric emergency departments.

### Rapid implementation of infection control strategies

Most departments used early clinical triage at the emergency department to separate suspected cases from other patients from the beginning of the survey period. Only one site (in Greece) initially did not triage but changed this in the last week of February. Two UK hospitals had plans to refer patients with positive test results to other hospitals for admission, and eight hospitals were planning to place multiple patients tested positive in cohort isolation if limited capacity for individual isolation occurred.

At most hospitals, staff used respirators, i.e. filtering face piece (FFP) masks, when treating suspected cases in the emergency department. In contrast, staff at four UK sites, in the Netherlands and at one site in Poland used surgical masks only. This did not change over the survey period.

## Discussion

In the early stages of the COVID-19 pandemic, paediatric emergency departments implemented standardised case definitions, testing guidelines and infection control measures rapidly. While this is an important and reassuring finding regarding the preparedness of paediatric emergency care in Europe, it may be a limitation of this survey that our sample of hospitals was biased towards tertiary care hospitals with strong international research links.

Although infection control strategies and even discharge of patients relied heavily on receiving SARS-CoV-2 test results, most hospitals only received these after considerable delay, often more than 24 h. Shortening turnaround times for tests should be a priority. Prior to discharge, infection control measures on uninfected patients awaiting test results place a huge burden on emergency care resources. Most departments rightly responded by discharging patients while test results were pending. This does not, however, mitigate against the public health impact of delayed result reporting on efficient contact tracing and subsequent isolation or quarantine of contacts in the community.

The guidelines for testing focused on two aims: establishing aetiology in children with symptoms of ARI and excluding infection for inpatient infection control purposes. This was a necessary restriction while numbers of new infections were high and capacities for testing were limited. We believe that in the current situation with vastly expanded laboratory capacities, a broader approach with more testing of mildly symptomatic patients or asymptomatic contacts may be warranted. To allocate testing resources responsibly, we believe that specific testing criteria for the paediatric population are needed because both the individual risk of children to suffer from severe disease and to sustain transmission in the community differ from that in adults [10, 11].

Children and adolescents suffer serious consequences from school closures and allowing schools to re-open has positive social, psychological and economic implications [12]. Benefits of broader access to testing may include the ability to detect outbreaks in day care facilities and schools earlier in order to limit spread of infections while maintaining as much normality as possible for children and adolescents.

## Abbreviations

A&E: Accident and emergency department
ARI: Acute respiratory infection
ECDC: European Centre for Disease Prevention and Control
HSCT: Haematopoietic stem cell transplantation
LRTI: Lower respiratory tract infection
PICU: Paediatric intensive care unit
SARS-CoV-2: Severe acute respiratory syndrome coronavirus 2
UK: United Kingdom
WHO: World Health Organization
